# HPLC Profile of Longan (cv. Shixia) Pericarp-Sourced Phenolics and Their Antioxidant and Cytotoxic Effects

**DOI:** 10.3390/molecules24030619

**Published:** 2019-02-11

**Authors:** Xuelian Bai, Rui Pan, Mingzhu Li, Xiuting Li, Huawei Zhang

**Affiliations:** 1 Beijing Advanced Innovation Center for Food Nutrition and Human Health, Beijing Technology and Business University (BTBU), Beijing 10048, China; baixl2012@163.com; 2 College of Life and Environmental Sciences, Hangzhou Normal University, Hangzhou 310036, China; 3 School of Pharmaceutical Sciences, Zhejiang University of Technology, Hangzhou 310014, China; yingzhoudengyuan@sina.com (R.P.); marinedrugs2017@163.com (M.L.)

**Keywords:** Longan pericarp, Shixia, polyphenol, HPLC, antioxidant, cytotoxicity

## Abstract

Longan (*Dimocarpus longan* Lour.) pericarp, the main by-product of aril and pulp processing, is abundant in phenolic compounds and worthy of further utilization. The present work firstly reported HPLC analysis and in vitro antioxidant evaluation of longan (cv. Shixia) pericarp-derived phenolics (LPPs), the purified longan pericarp extract (LPE), as well as their cytotoxic effect on lung cancer cell line, A549. The results indicated that the purified LPE had significant amounts of phenolics, with content of 57.8 ± 0.6 mg of gallic acid equivalents per gram of dry longan pericarp (mg GAE·g^−1^ DLP), which consisted of six phenolic compounds (A–F), including protocatechuic acid (A), isoscopoletin (B), quercetin (C), ellagic acid (D), corilagin (E), and proanthocyanidins C1 (F). Antioxidant assays showed that LPPs (10 μM) and LPE (1.0 mg·mL^−1^) had certain antioxidant activities, in which corilagin (E) possessed the best DPPH radical scavenging rate 71.8 ± 0.5% and •OH inhibition rate 75.9 ± 0.3%, and protocatechuic acid (A) exhibited the strongest Fe^2+^ chelating ability 36.4 ± 0.7%. In vitro cytotoxic tests suggested that LPPs had different effect on A549 cell line, in which corilagin (E) exhibited potent cytotoxicity with an IC_50_ value of 28.8 ± 1.2 μM. These findings were further confirmed by cell staining experiments.

## 1. Introduction

It is well-known that polyphenols derived from edible fruit not only have a variety of biological activities [1,2], but also possess potential to confer positive gut health benefits [3]. Longan (*Dimocarpus longan* Lour.), one of the most popular fruits in temperate and sub-tropical regions, has a succulent and edible aril, with delicious flavor and health effects, as well as pharmacological properties, such as treating or relieving insomnia, neural pain, swelling, hyperglycaemia, improving women’s health after giving birth to a child and increasing the immunomodulatory capacity [4,5,6,7]. These therapeutic benefits are partially ascribed to their unique phytochemical ingredients, including polyphenols.

China has been recognized as the origin of longan, where cultivation has a long history of more than 2000 years and approximately 300 cultivars have been selected for production [8]. In 2015, the total production of longan fruit was over 1.8 million tons in China [9]. Longan pericarp and seed, as by-products of aril and pulp processing, account for 16–40% of the whole fruit by weight [6]. Fresh longan pulp has rich nutrient substances, such as water (81.4%), total carbohydrate (12.38–22.55%), vitamin K (196.5 mg·100 g^−1^), ascorbic acid (43.12–163.7 mg·100 g^−1^) [6]. Furthermore, a growing number of evidence indicates that pericarp of mature longan fruit is rich in phenolic compounds, with a broad spectrum of bioactive properties [10,11,12,13]. Some longan pericarp-derived polyphenols had been detected and identified using chromatographic and spectroscopic methods, such as 4-*O*-methylgallic acid and (-)-epicatechin from *D. longan* Lour. cv. Shixia [10], corilagin from *D. longan* Lour. (unknown cv.) [12], and 17 phenolic compounds from *D. longan* Lour. cv. Wulongling, including gallic acid, ellagic acid, epicatechin polymers, procyanidin B2, rutin [13]. Shixia is one of the most popular longan cultivars and holds first or second output of over 0.3 million tons in China [14]. However, there is no literature about its pericarp-derived phenol composition and related bioactivity. In order to better understand and make use of ‘Shixia’ longan pericarp, therefore, the extraction and HPLC analysis of longan pericarp phenols (LPPs) were firstly carried out in this work, as well as the evaluation of their antioxidant activities and cytotoxic effects on human lung carcinoma A549 cell line.

## 2. Results and Discussion

### 2.1. Total Phenolic Content and HPLC Analysis

Microwave and ultrasonic-assisted extraction technique was successfully employed to extract phenol compounds from fresh lyophilized longan (cv. Shixia) percarp with 60% aqueous ethanol, owing to its higher yield of total phenolic extraction by comparison with conventional approaches [6,15]. Then the crude longan pericarp extract (LPE) was subjected to purification with resin to afford phenol-enriched LPE. The amount of total phenolics in the purified LPE was determined to be 57.8 ± 0.6 mg of gallic acid equivalents per gram of dry longan pericarp (mg GAE·g^−1^DLP) using the modified Folin–Ciocalteu procedure [16,17].

**Figure 1:**
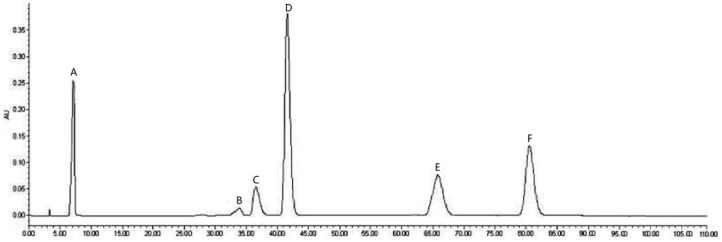
HPLC chromatogram of purified longan pericarp extract (LPE) at 280 nm.

**Figure 2:**
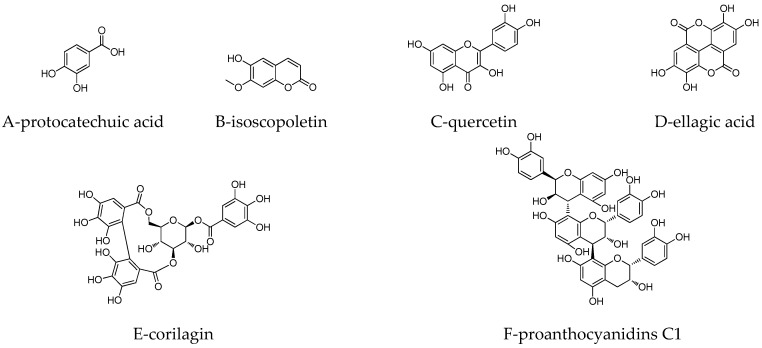
Chemical structures of LPPs in purified LPE.

As shown in Figure 1, six compounds were detected in the purified LPE by HPLC technique. Using external standard methods and spectral analysis of HRESI-MS and ^1^H NMR (Figures S1–S12), these LPPs were unambiguously identified and respectively characterized as proto-catechuic acid (A), isoscopoletin (B), quercetin (C), ellagic acid (D), corilagin (E), and proanthocyanidins C1 (F) (Figure 2). The results of the quantitative analysis indicated that the principal component in the purified LPE was ellagic acid (17.37 ± 0.32 mg·kg^−1^ DLP), followed by proanthocyanidins C1 (9.77 ± 0.45 mg·kg^−1^ DLP), corilagin (5.25 ± 0.61 mg·kg^−1^ DLP), protocatechuic acid (5.14 ± 0.52 mg·kg^−1^ DLP), quercetin (3.12 ± 0.76 mg·kg^−1^ DLP), and isoscopoletin (0.92 ± 0.13 mg·kg^−1^ DLP).

These findings suggested that the total phenolic content and each component in the purified extract of longan (cv. Shixia) pericarp were not identical, but similar with several previous studies, due to various cultivar, degree of ripeness, and preparation method. Jiang and his coworkers compared the yields of high pressure-assisted extraction (HPE) and conventional extraction (CE), using 50% ethanol as solvent [15]. The results indicated that HPE had a higher yield of total phenolic content (20.8 ± 1.6 mg GAE·g^−1^ DLP) in the crude extract of longan (cv. Shixia) pericarp than CE, which yield was 14.6 ± 0.2 mg GAE·g^−1^ DLP. As far as its phenolic components are concerned, only three phenolic compounds, namely gallic acid, corilagin, and ellagic acid, were identified by external standard method and each content ranged from 0.1 to 10 mg·g^−1^ DLP. Huang et al. also obtained the polyphenol-enriched extract from longan (unknown cultivar) pericarp, using conventional extraction method and methanol as solvent, with a much higher yield of about 378 mg GAE·g^−1^ DLP [18]. 

### 2.2. Antioxidant Activity of LPPs and Purified LPE

In order to explore the antioxidant activity of LPPs and the purified LPE, their scavenging DPPH and OH radical rates, and chelating ferrous ion capacity were evaluated. As shown in Table 1, the purified LPE had certain antioxidant activity at 1.0 mg·mL^−1^, and these LPPs exhibited different antioxidant capacity at 10 μM. These results indicated that corilagin (E) possessed the best DPPH• scavenging rate (71.8 ± 0.5%) and •OH inhibition rate (75.9 ± 0.3%), and protocatechuic acid (A) had the strongest chelation activity of ferrous ion (36.4 ± 0.7%). These results were consistent with the results reported before [19,20]. Meanwhile, proanthocyanidins C1 (F) exhibited the weakest capacity of scavenging DPPH radical and chelating ferrous ion, and quercetin (C) had a minimum •OH inhibition rate.

### 2.3. Cytotoxic Effect of LPPs and Purified LPE

**Figure 3:**
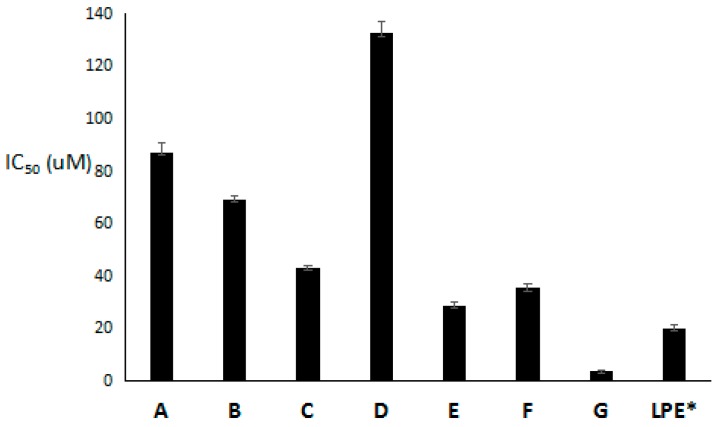
Inhibitory effect of LPPs and purified LPE on A549 cells. (A-protocatechuic acid, B-isoscopoletin, C-quercetin, D-ellagic acid, E-corilagin, F-proanthocyanidins C1, G-5-fluorouracil, LPE-longan pericarp extract, * 20.0 ± 1.5 mg·mL^−1^).

In vitro bioassay results suggested that LPPs and the purified LPE possessed certain cytotoxic effects on lung cancer A549 cell line to a different extent (Figure 3). The purified LPE had an IC_50_ value of 20.0 ± 1.5 mg·mL^−1^, while corilagin (E) exhibited the strongest inhibitory effect with an IC_50_ value of 28.8 ± 1.2 μM. Structurally, corilagin is a member of the tannin family that had been discovered in many medicinal plants and has been shown to possess potential cytotoxic activity against ovarian cancer cell lines SKOv3ip and Hey, with IC_50_ values of less than 30 μM [21,22]. And other LPPs, protocatechuic acid, isoscopoletin, quercetin, ellagic acid and proanthocyanidins C1 had weak cytotoxicities with IC_50_ values range from 35.3 ± 1.2 to 132.5 ± 4.5 μM.

**Figure 4:**
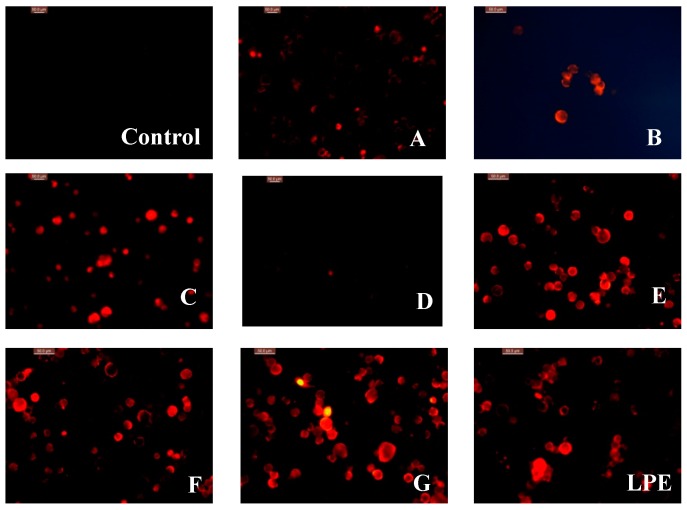
Fluorescence microscopy of A549 cell line treated with LPPs and purified LPE. (Control-untreated, **A**-protocatechuic acid, **B**-isoscopoletin, **C**-quercetin, **D**-ellagic acid, **E**-corilagin, **F**-proanthocyanidins C1, **G**-5-fluorouracil, LPE-Longan pericarp extract).

These findings were confirmed by observing the morphological variation of the nucleus of A549 cell line, using PI (3,8-diamino-5-[3-(diethylmethylammonio)propyl]-6-phenyl-phenanthridinium diiodide) staining (Figure 4). The strong red fluorescence of the cell nucleus treated with corilagin, quercetin, proanthocyanidins C1 and the purified LPE, indicated that they had potent in vitro cytotoxic effects. While the weak fluorescence showed that protocatechuic acid, isoscopoletin, and ellagic acid possessed less potent inhibitory activity against A549 cells.

## 3. Experimental Section

### 3.1. General Experimental Procedure

All NMR experiments were run on a Bruker Avance 600 MHz spectrometer equipped with a 5-mm triple resonance (HCN) cold probe. Positive HR-ESI-MS spectra were record on a Bruker micrOTOF-Q II mass spectrometer. Acetonitrile, formic acid and water used in HPLC system were of chromatographic grade and other chemicals were analytical.

### 3.2. Preparation of Purified LPE

Mature longan (D. longan Lour. cv. Shixia) was grown in Foshan (China) and purchased from fruit market in Hangzhou City (China). The pericarp was manually separated from the whole fruit and freeze-dried (FD-A10N-50, Shanghai Kuansons Instrument Co., Ltd., China). Dry longan pericarp (DLP) was ground and then passed through a 40 mesh sieve. About 50 g of DLP powder was extracted, with 500 mL of 60% aqueous ethanol, using a microwave and ultrasonic-assisted extractor (CW-2000, Xintuo Microwave Equipment Co., Ltd., Shanghai, China) for 20 min. The obtained LPE was concentrated under reduced pressure and lyophilized (RE-3000A, Shanghai Yarong Instrument Co., Ltd., China). After re-dissolving in 500 mL of 20% ethanol solution, the resulting extract was centrifuged for 20 min at 50,000× *g* (Centrifuge 5810R, Eppendorf Co., Ltd., Germany) and retained on a normal atmosphere column with 500 mL macro-porous absorbent resin D101 (Tianjin Yunkai Co., Ltd., China), followed by eluting with three bed volumes of 80% alcohol. The collected elution was concentrated under reduced pressure and lyophilised. The afforded phenol-enriched extract was dissolved in methanol, with a final concentration of 10 mg·mL^−1^ and preserved at 4 °C before HPLC analysis.

### 3.3. Determination of Total Phenolic Content

The total phenolic content of purified LPE was determined, using the modified Folin–Ciocalteu approach [16,17]. Two mL of sodium carbonate (20%, *w*/*v*) was added to samples with different concentrations in a 10-mL volumetric flask. After 5 min, 0.1 mL of Folin-Ciocalteu reagent (Sigma-Aldrich Chemical Co., St. Louis, MO, USA) was added, and the volume was increased to 10 mL, with deionized H_2_O. After incubation at 30 °C for 1 h, the absorbance was recorded at 750 nm on a Hitachi-UV-3000 spectrometer (Hitachi, Tokyo, Japan), and compared to a gallic acid calibration curve. Triplicate tests were conducted for each sample and the total phenolic content was expressed as milligram of gallic acid equivalents per gram of DLP (mg GAE·g^−1^ DLP).

### 3.4. HPLC Analysis of LPPs

Six phenolic standards including protocatechuic acid (A), isoscopoletin (B), quercetin (C), ellagic acid (D), corilagin (E) and proanthocyanidins C1 (F), and the positive control 5-fluorouracil were purchased from Sigma-Aldrich Chemical (St. Louis, MO, USA) and respectively dissolved in methanol with the initial concentration of 1.0 mM. HPLC analysis of LPPs was performed using a HPLC system (Waters Alliance 2695, Massachusetts, USA) equipped with a 717plus auto-sampler and a 2487 UV-visible detector on a HPLC column (XTerra RP-8, 250 mm × 4.6 mm, 5 µm) (Waters, Milford, MA). The detection wavelength was set at 280 nm. The temperature of column was maintained at 20 °C. A gradient solvent system consisting of solvent A (0.1% aqueous formic acid) and solvent B (acetonitrile) was used at 0.5 mL·min^−1^ as following gradient procedure: 0–25 min, 10 to 25% B; 25–50 min, 25% B; 50–70 min, 25 to 30% B; 70–90 min, 30 to 100% B; 90–100 min, 100% B; 100–110 min, and 100–10% B. All solvents were filtered with a 0.45 µm membrane filter and each injection volume was 5 µL. Quantitative analysis of LPPs was carried out using the external standard method described before [21]. Triplicate tests were conducted for each sample. The level of each compound was expressed in mg·kg^−1^ DLP.

### 3.5. Antioxidant Assay of LPPs and Purified LPE

#### 3.5.1. DPPH Radical Scavenging Assay

To assess the radical scavenging capacity of LPPs and the purified LPE, DPPH radical scavenging activity was investigated according to the procedure reported by Villaño et al. [23,24]. 100 μL of sample solution (10 μM) or LPE (1.0 mg·mL^−1^) was added to 100 μL of 0.2 mM DPPH in a 96-well plate, then incubated in the dark room temperature (25 °C) for 30 min. The absorbance of the mixture was measured at 517 nm by enzyme-linked immunosorbent assay plate reader (Multiskan Sky, Thermo Electron Co., Waltham, MA, USA). DPPH radical scavenging activity was calculated as follows:

(1) $$Scavenging\;rate=\frac{\left(A_{0}-A_{1}\right)}{A_{0}}\times 100\%$$

where *I*_DPPH_ is the DPPH radical scavenging rate (%), *A*_0_ is the absorbance of a negative control, *A*_1_ is the absorbance of each sample. All measurements were performed in triplicate.

#### 3.5.2. Hydroxyl Radical (•OH) Scavenging Assay

Hydroxyl radical (•OH) assay was performed according to the procedure reported by Sun et al. [25,26]. The reaction mixture contained 1.0 mL of LPP (10 μM) or the purified LPE (1.0 mg·mL^−1^) was incubated with 1.0 mL phenanthroline (2.5 mM), 1.0 mL distilled water, 1.0 mL ferrous sulfate (2.5 mM) and 1.0 mL hydrogen peroxide (20 mM) in phosphate buffer (20 mM, pH 7.4) for 90 min at 37 °C. The absorbance was measured on a spectrophotometer (UV-3000, Hitachi Co., Ltd., Tokyo, Japan) at 536 nm. The ability to scavenge hydroxyl radical was calculated using the following equation:

(2) $$Inhibition\;rate\left(\%\right)=\frac{A_{3}-A_{1}}{A_{2}-A_{1}}\times 100\%$$

where *A*_1_ is the absorbance of reaction mixture of hydrogen peroxide (H_2_O_2_), *A*_2_ is the absorbance of reaction mixture of distilled water, and *A*_3_ is the absorbance of reaction mixture of each sample. All measurements were performed in triplicate.

#### 3.5.3. Ferrous Ion Chelating Assay

Ferrous ion chelating capability was measured with the method described by Wang et al. [27]. A quantity of 2.0 mL of LPP (10 μM) or LPE (1.0 mg·mL^−1^) was mixed with 100 mL FeCl_2_ (ferrous chloride, 2 mM) and 1.7 mL deionized water. Then, 200 μL of ferrozine (5 mM) was added into the mixture, and fully mixed. The solution was then incubated at 25 °C for 10 min. The absorbance was measured using a spectrometer (UV-3000, Hitachi Co., Ltd., Tokyo, Japan) at 562 nm with ethylenediamine tetracetic acid disodium salt (EDTA-2Na) as a positive control. Ferrous ion chelating activity was calculated as follows:

(3) $$Ferrousionchelatingactivity\left(\%\right)=\left[1-\frac{A_{1}-A_{2}}{A_{0}}\right]\times 100\%$$

where *A*_0_ is the absorbance of the control (deionized water instead of sample); *A*_1_ is the absorbance of the sample; *A*_2_ is the absorbance of the sample only (deionized water instead of ferrous chloride). All measurements were performed in triplicate.

### 3.6. Cytotoxic Effect of LPPs and Purified LPE on Cell Line A549

#### 3.6.1. Inhibitory Activity

Human NSCLC A549 cell line was obtained from Cancer Research Center of Hangzhou Normal University (Hangzhou, China). Firstly, these cells were maintained in RPMI-1640 (Yuanye Biotech Co., Ltd., Shanghai, China) medium consists of 10% FBS (Yuanye Biotech Co., Ltd., Shanghai, China) and 1% (0.01 g·mL^−1^) penicillin-streptomycin (Sigma-Aldrich, St. Louis, MO) followed by incubation at 37 °C in a humidified 5% CO_2_ incubator (Chengdu Must Bio-Technology Co., Ltd., Chengdu, China). The inhibitory effect of LPPs and LPE on cell proliferation was performed according to our previous report [16]. Briefly, cells in 96-well plates at a density of 5 × 10^3^ per well were treated for 24 h with chemicals at different doses (6.25, 12.5, 25, 50, 75, 100, 150, 200 μM) or the purified LPE at different concentrations (5, 10, 20, 40, 80, and 100 mg·mL^−1^) with 5-fluorouracil as the positive control. Each well was added with 10 μL of cell counting kit-8 (CCK-8) solution (Shenggong Bioengineering Co., Ltd., Shanghai, China), and the cells were further incubated for 1 h. Then the medium containing CCK-8 was removed and 100 μL of DMSO was added to each well. The plate was gently shaken for 15 min to dissolve the formazan crystals (Sigma-Aldrich, St. Louis, MO, USA) and the absorbance was measured at 450 nm on a ELISA (Multiskan Sky, Thermo Electron Co., MA, USA). The percentage viability was calculated using the following formula:

(4) $$IC=\left[1-\frac{OD_{sample}-OD_{blank}}{OD_{negative}-OD_{blank}}\right]\times 100\%$$

where *IC* is the percentage viability, *OD_sample_* is the absorbance of sample group (10 µL CCK-8 reagent + 10 µL sample solution + 90 µL cell suspension), *OD_negative_* is the absorbance of (10 µL CCK-8 reagent + 10 µL cell medium + 90 µL cell suspension), *OD_blank_* is the absorbance of blank group (10 µL CCK-8 reagent + 10 µL sample solution + 90 µL cell medium). The concentration of LPPs and the purified LPE needed to inhibit cell growth by 50% (IC_50_) was calculated from the dose response curves for each cell line [28]. All measurements were performed in triplicate.

#### 3.6.2. Fluorescence Staining of PI

The logarithmic phase of A549 cells were washed with phosphate buffered saline (PBS) and digested with trypsin. When reaching a density of 5 × 10^4^ CFU per mL, the cells were added with 1 mL sample solution or DMSO. Then the cells were added with 1 mL culture medium and incubated at 37 °C for 24 h. The cells treated with sterile PSB washing, with trypsin digestion cells, using RPMI 1640 culture medium containing 10% fetal bovine serum termination of digestion, made the cell suspension, the cell suspension put in centrifuge tube, 1000 r/min, the centrifugal 5 min, abandon supernatant. Plus 5 μL PI (0.2 mg·mL^−1^) (Sigma-Aldrich, St. Louis, MO, USA) and kept in dark at 37 °C for 30 min, after the incubation, washing with buffer, add 1 mL culture [29,30]. All images were taken by an inverted fluorescence microscopy (Leica Co., Wetzlar, Germany).

### 3.7. Statistical Analysis

All values were given as means ± standard and analyzed by SPSS software (Version 23.0, Chicago, IL, USA). A value of *p* < 0.05 were accepted as significant.

## 4. Conclusions

The present work firstly reported on HPLC analysis and antioxidant activities of the purified LPE and LPPs as well as their cytotoxic effect on A549 cell line. These results suggested that the purified LPE is rich in phenolic compounds including protocatechuic acid (A), isoscopoletin (B), quercetin (C), ellagic acid (D), corilagin (E) and proanthocyanidins C1 (F). The antioxidant assay suggested that the purified LPE had certain capacity of scavenging DPPH•, inhibiting OH radical, and chelating ferrous ion. Compound E was shown to be the best antioxidant agent with DPPH• scavenging rate (71.8 ± 0.5%) and OH• inhibition rates (75.9 ± 0.3%) at 10 μM. And compound A possessed the strongest Fe^2+^ chelating ability (36.4 ± 0.7%). In vitro cytotoxic evaluation indicated that corilagin (E) also exhibited the strongest inhibitory effect on the growth of A549 cells, with an IC_50_ value of 28.8 ± 1.2 μM. Fluorescence staining experiments also confirmed that compounds E and F possess potent anti-proliferative activity against A549 cell. These findings would improve comprehensive utilization of longan (cv. Shixia) pericarp to make value-added products, including antioxidant food and pharmaceutical agents.

## Figures and Tables

**Table 1:** Antioxidant activities of longan pericarp phenols (LPPs) and purified longan pericarp extract (LPE) (*n* = 3).

	Compound	Antioxidant Activity (%)		
		DPPH• Scavenging Rate	•OH Inhibition Rate	Ferrous Ion Chelation Effect
A *	protocatechuic acid	59.6 ± 0.5	56.9 ± 0.5	36.4 ± 0.7
B *	isoscopoletin	48.9 ± 0.8	43.2 ± 0.1	28.9 ± 0.2
C *	quercetin	53.6 ± 0.4	18.3 ± 0.5	22.3 ± 0.5
D *	ellagic acid	45.6 ± 0.4	51.3 ± 0.5	33.1 ± 0.2
E *	corilagin	71.8 ± 0.5	75.9 ± 0.3	32.3 ± 0.5
F *	proanthocyanidins C1	28.8 ± 0.1	36.9 ± 0.2	5.3 ± 0.4
LPE ^※^	longan pericarp extract	55.5 ± 0.3	60.2 ± 0.3	23.9 ± 0.8

(* 10 μM, ^※^ 1.0 mg·mL^−1^).

## Supplementary Materials

The Following are available online, Figures S1–S12: ^1^H NMR and HR-ESI-MS spectra for longan pericarp-derived phenolics A–F are available online.
