# Supplementary material for: HPLC Profile of Longan (cv. Shixia) Pericarp-Sourced Phenolics and Their Antioxidant and Cytotoxic Effects
Source: Molecules. 2019 Feb 11;24(3):619. doi: 10.3390/molecules24030619 (PMC6384674; doi:10.3390/molecules24030619)
Supplement: Supplementary file 1 [file molecules-24-00619-s001.pdf]

Supplementary Materials

HPLC profile of longan pericarp-sourced phenolics and their antioxidant and cytotoxic effects

Xuelian Bai, Rui Pan, Mingzhu Li, Xiuting Li *, Huawei Zhang *

Table of Contents

Figure S1	^{1}H NMR spectrum of A (600 MHz, in MeOH-d_4)	S2
Figure S2	HR-ESI-MS spectrum of A	S3
Figure S3	^{1}H NMR spectrum of B (600 MHz, in MeOH-d_4)	S4
Figure S4	HR-ESI-MS spectrum of B	S5
Figure S5	^{1}H NMR spectrum of C (600 MHz, in MeOH-d_4)	S6
Figure S6	HR-ESI-MS spectrum of C	S7
Figure S7	^{1}H NMR spectrum of D (600 MHz, in DMSO-d_6)	S8
Figure S8	HR-ESI-MS spectrum of D	S9
Figure S9	^{1}H NMR spectrum of E (600 MHz, in MeOH-d_4)	S10
Figure S10	HR-ESI-MS spectrum of E	S11
Figure S11	^{1}H NMR spectrum of F (600 MHz, in MeOH-d_4)	S12
Figure S12	HR-ESI-MS spectrum of F	S13

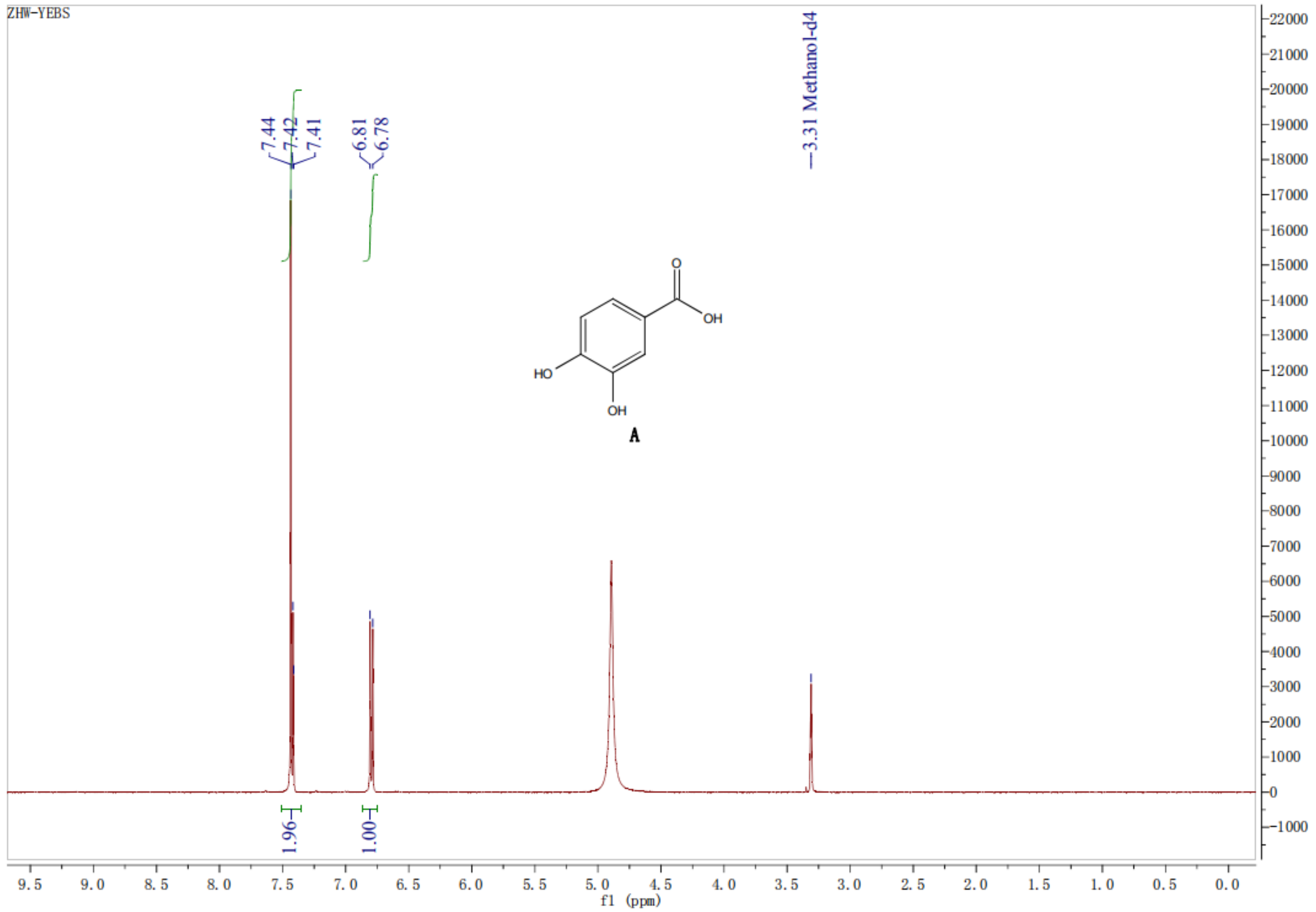

Figure S1 ^{1}H NMR spectrum of A (600 MHz, in MeOH-d_4)

Mass Spectrum SmartFormula Report

Analysis Info

Analysis Name	D:\Data\2018\1220\A..d	Acquisition Date	12/20/2018 1:44:14 PM
Method	tune_pos_low2.m	Operator	BDAL@DE
Sample Name	A	Instrument / Ser#	micrOTOF-Q II 10366
Comment			

Acquisition Parameter

Source Type	ESI	Ion Polarity	Positive	Set Nebulizer	0.3 Bar
Focus	Active	Set Capillary	4800 V	Set Dry Heater	180 °C
Scan Begin	100 m/z	Set End Plate Offset	-500 V	Set Dry Gas	4.0 l/min
Scan End	1000 m/z	Set Collision Cell RF	300.0 Vpp	Set Divert Valve	Waste

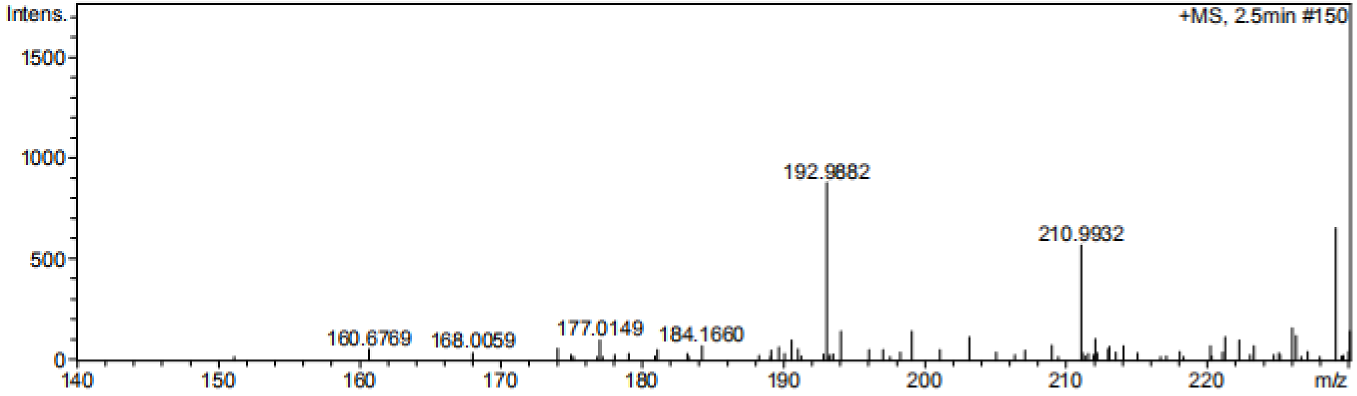

Meas. m/z	Formula	m/z	err [ppm]	mSigma
192.9882	C 7 H 6 K O 4	192.9898	8.2	64.4

Figure S2 HR-ESI-MS spectrum of A

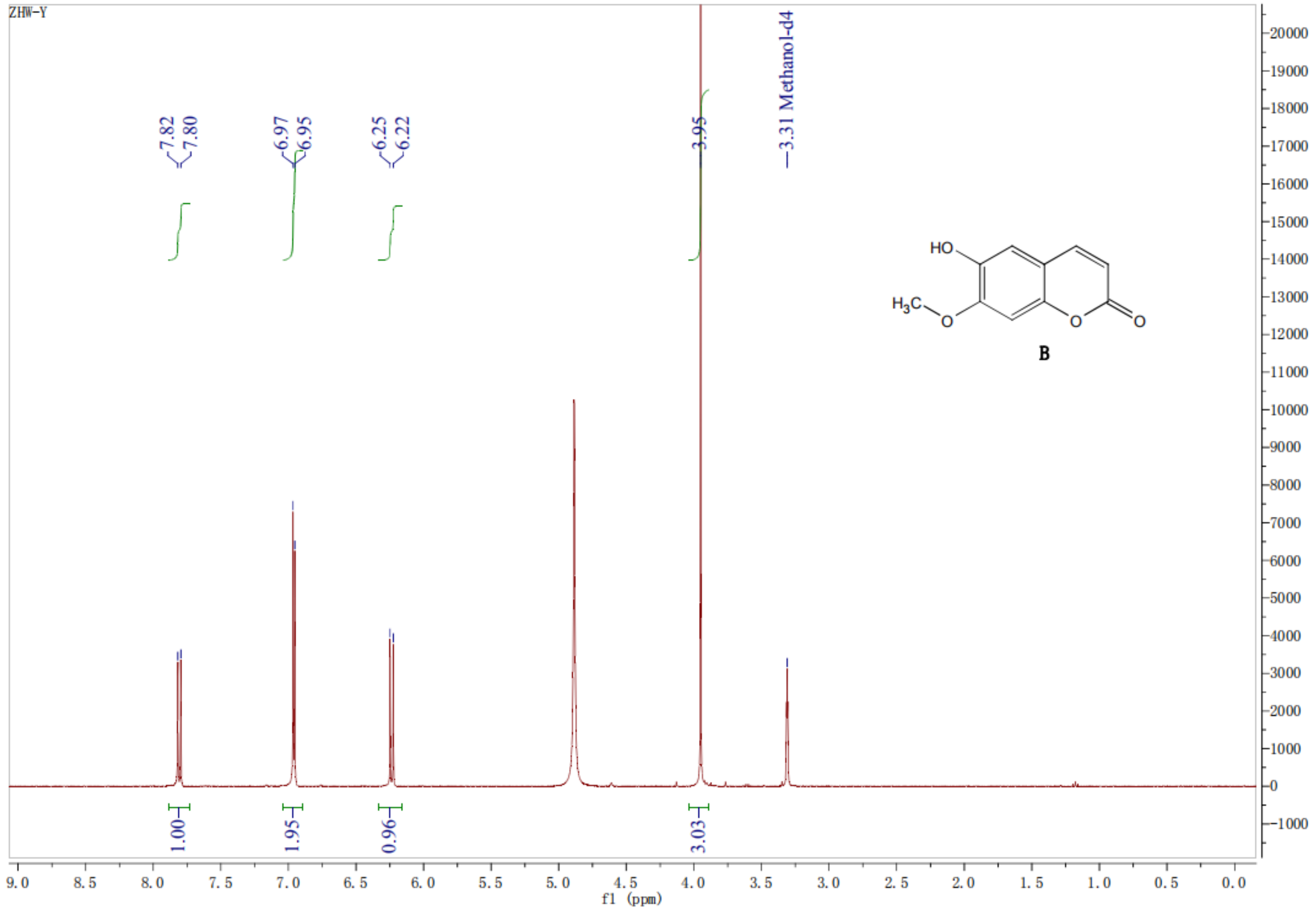

Figure S3 ^{1}H NMR spectrum of B (600 MHz, in MeOH-d_4)

Mass Spectrum SmartFormula Report

Analysis Info

Analysis Name	D:\Data\2018\1220\B.d	Acquisition Date	12/20/2018 10:09:56 AM
Method	tune_pos_low2.m	Operator	BDAL@DE
Sample Name	B	Instrument / Ser#	micrOTOF-Q II 10366
Comment			

Acquisition Parameter

Source Type	ESI	Ion Polarity	Positive	Set Nebulizer	0.3 Bar
Focus	Active	Set Capillary	4800 V	Set Dry Heater	180 °C
Scan Begin	100 m/z	Set End Plate Offset	-500 V	Set Dry Gas	4.0 l/min
Scan End	1000 m/z	Set Collision Cell RF	300.0 Vpp	Set Divert Valve	Waste

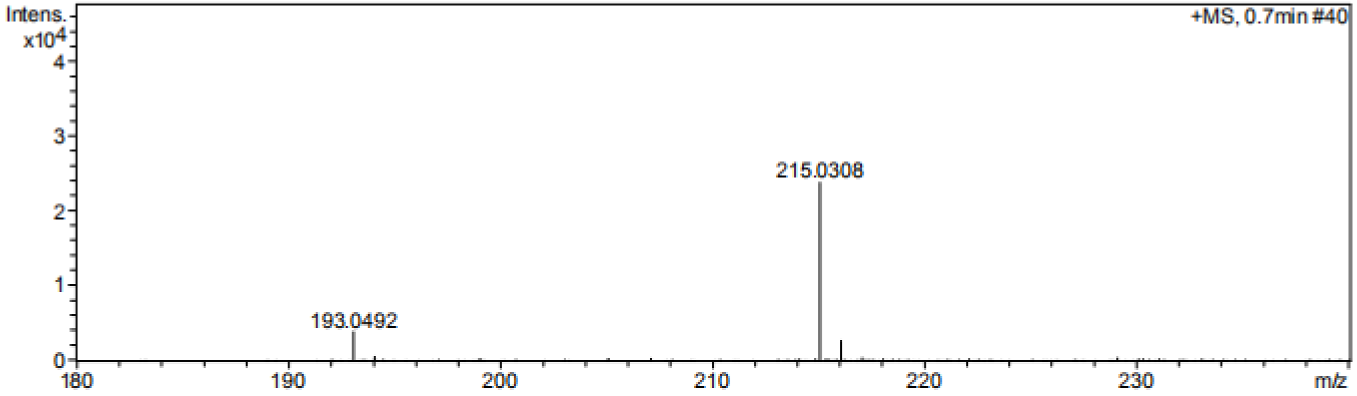

Meas. m/z	Formula	m/z	err [ppm]	mSigma
215.0308	C 10 H 8 Na O 4	215.0315	3.2	2.5

Figure S4 HR-ESI-MS spectrum of B

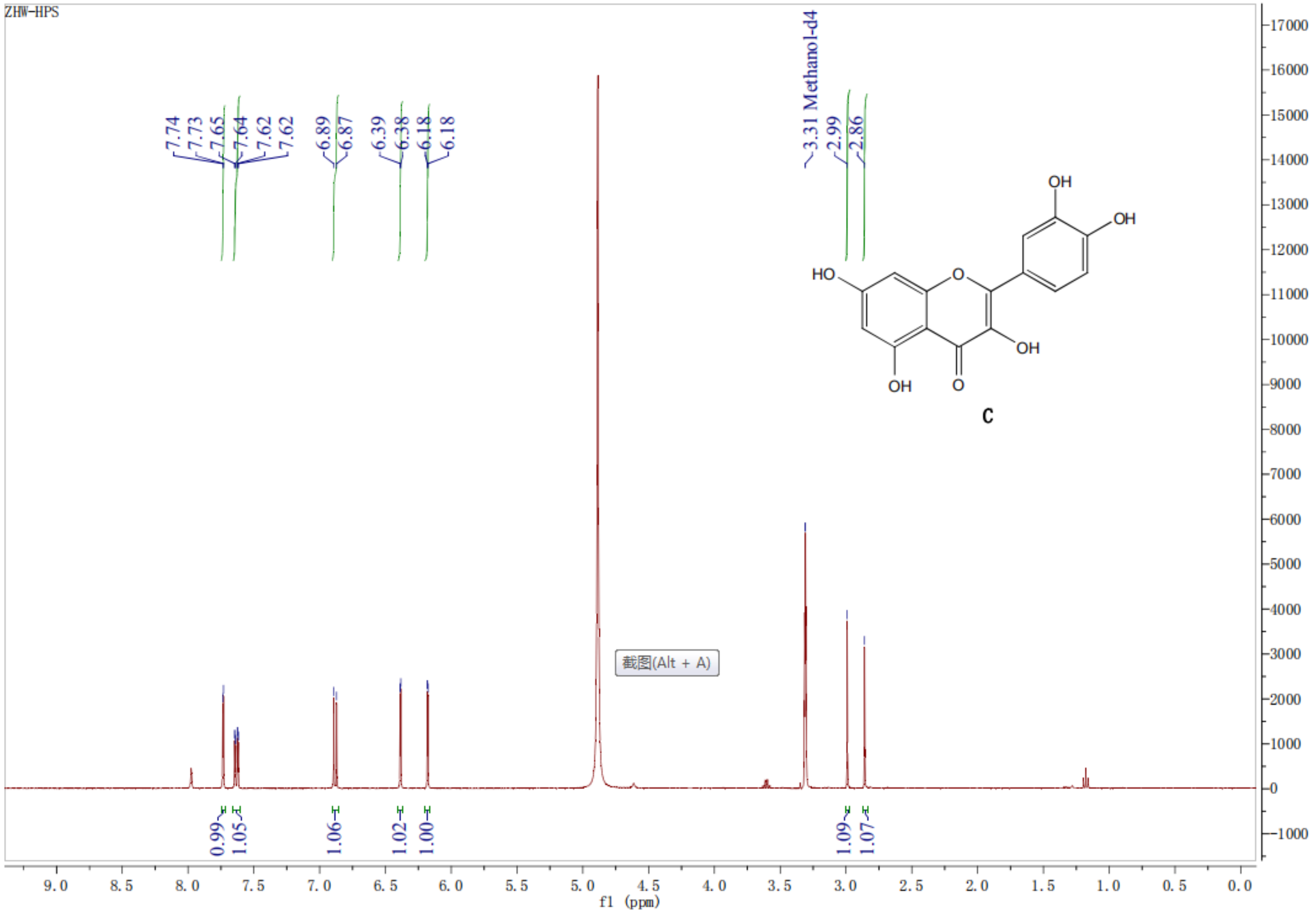

Figure S5 ^{1}H NMR spectrum of C (600 MHz, in MeOH-d_4)

Mass Spectrum SmartFormula Report

Analysis Info

Analysis Name	D:\Data\2018\1220\C.d	Acquisition Date	12/20/2018 10:12:22 AM
Method	tune_pos_low2.m	Operator	BDAL@DE
Sample Name	C	Instrument / Ser#	micrOTOF-Q II 10366
Comment			

Acquisition Parameter

Source Type	ESI	Ion Polarity	Positive	Set Nebulizer	0.3 Bar
Focus	Active	Set Capillary	4800 V	Set Dry Heater	180 °C
Scan Begin	100 m/z	Set End Plate Offset	-500 V	Set Dry Gas	4.0 l/min
Scan End	1000 m/z	Set Collision Cell RF	300.0 Vpp	Set Divert Valve	Waste

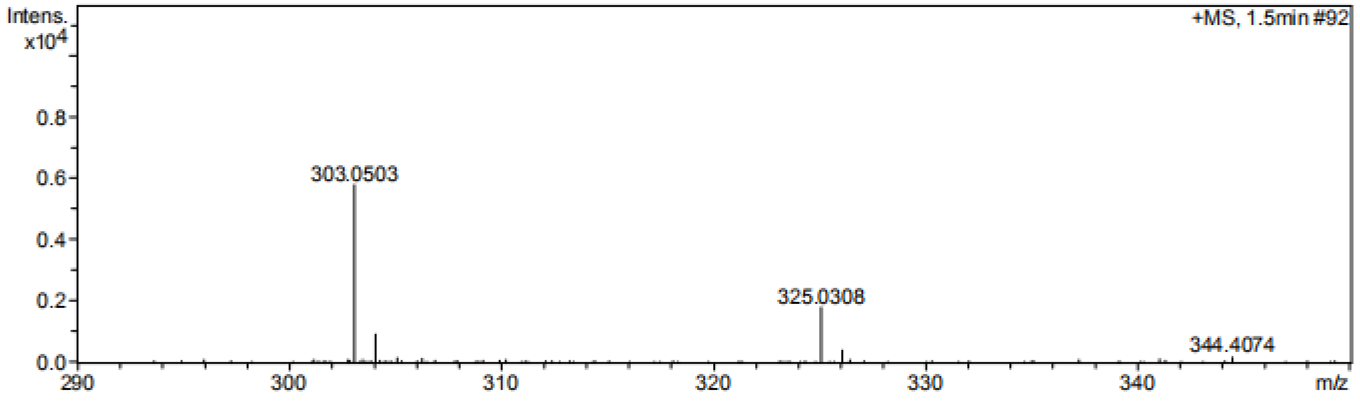

Meas. m/z	Formula	m/z	err [ppm]	mSigma
303.0503	C 15 H 11 O 7	303.0499	-1.1	3.1

Figure S6 HR-ESI-MS spectrum of C

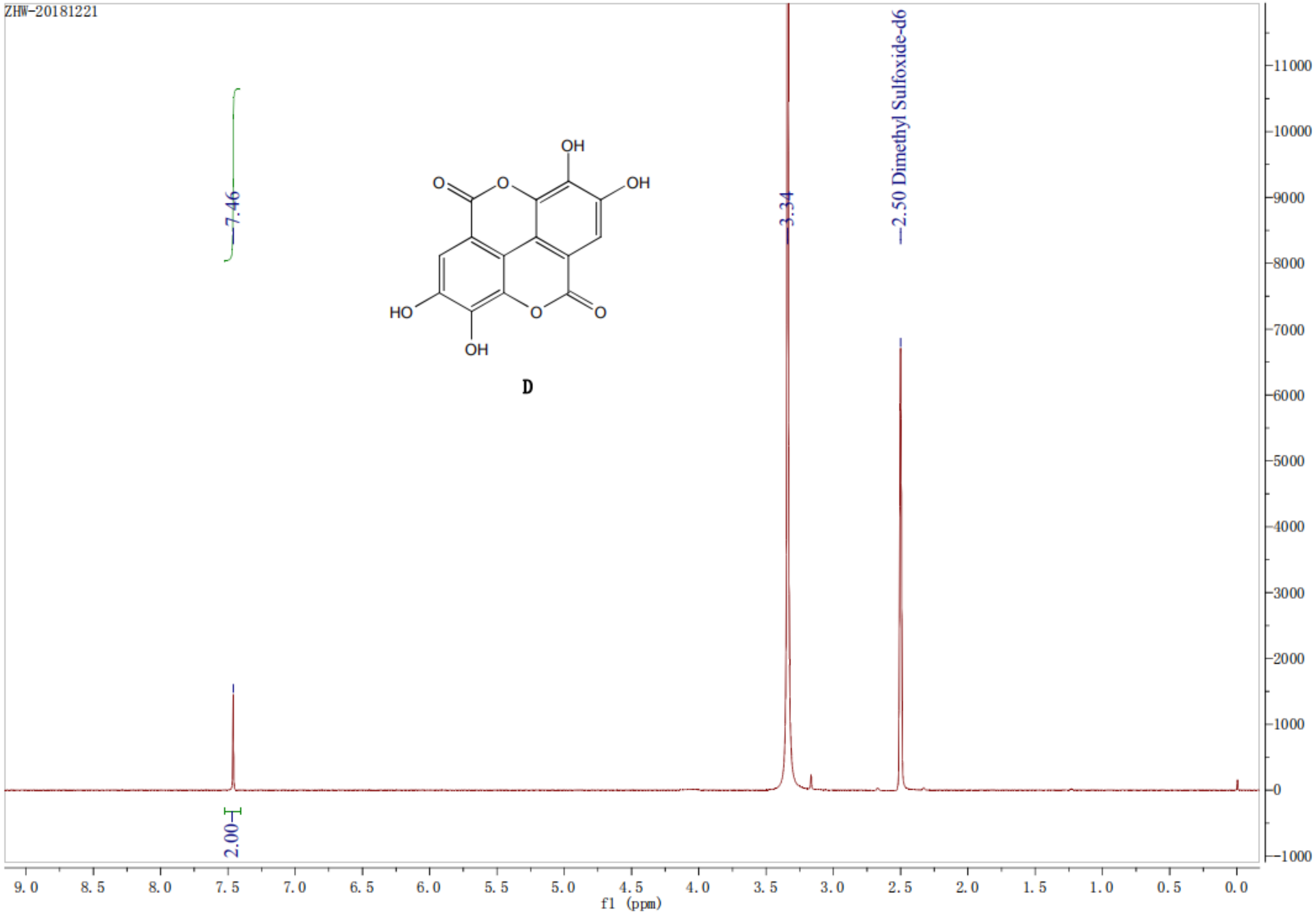

Figure S7 ^{1}H NMR spectrum of D (600 MHz, in DMSO-d_6)

Mass Spectrum SmartFormula Report

Analysis Info

Analysis Name	D:\Data\2018\1222\G..d	Acquisition Date	12/22/2018 3:00:38 PM
Method	tune_pos_low2.m	Operator	BDAL@DE
Sample Name	G	Instrument / Ser#	micrOTOF-Q II 10366
Comment			

Acquisition Parameter

Source Type	ESI	Ion Polarity	Positive	Set Nebulizer	0.3 Bar
Focus	Active	Set Capillary	4900 V	Set Dry Heater	180 °C
Scan Begin	100 m/z	Set End Plate Offset	-550 V	Set Dry Gas	4.0 l/min
Scan End	1000 m/z	Set Collision Cell RF	300.0 Vpp	Set Divert Valve	Waste

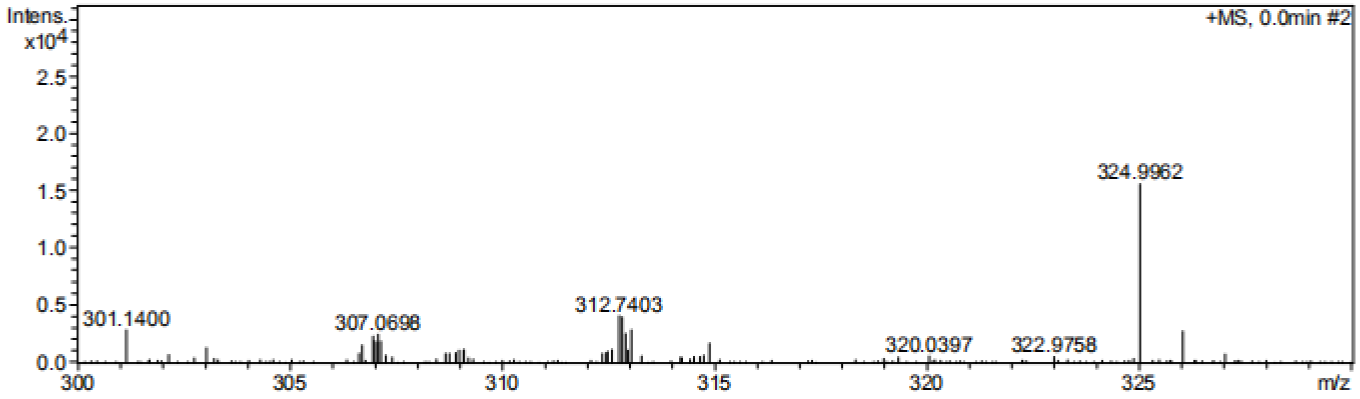

Meas. m/z	Formula	m/z	err [ppm]	mSigma
324.9962	C 14 H 6 Na O 8	324.9955	-2.2	17.9

Figure S8 HR-ESI-MS spectrum of D

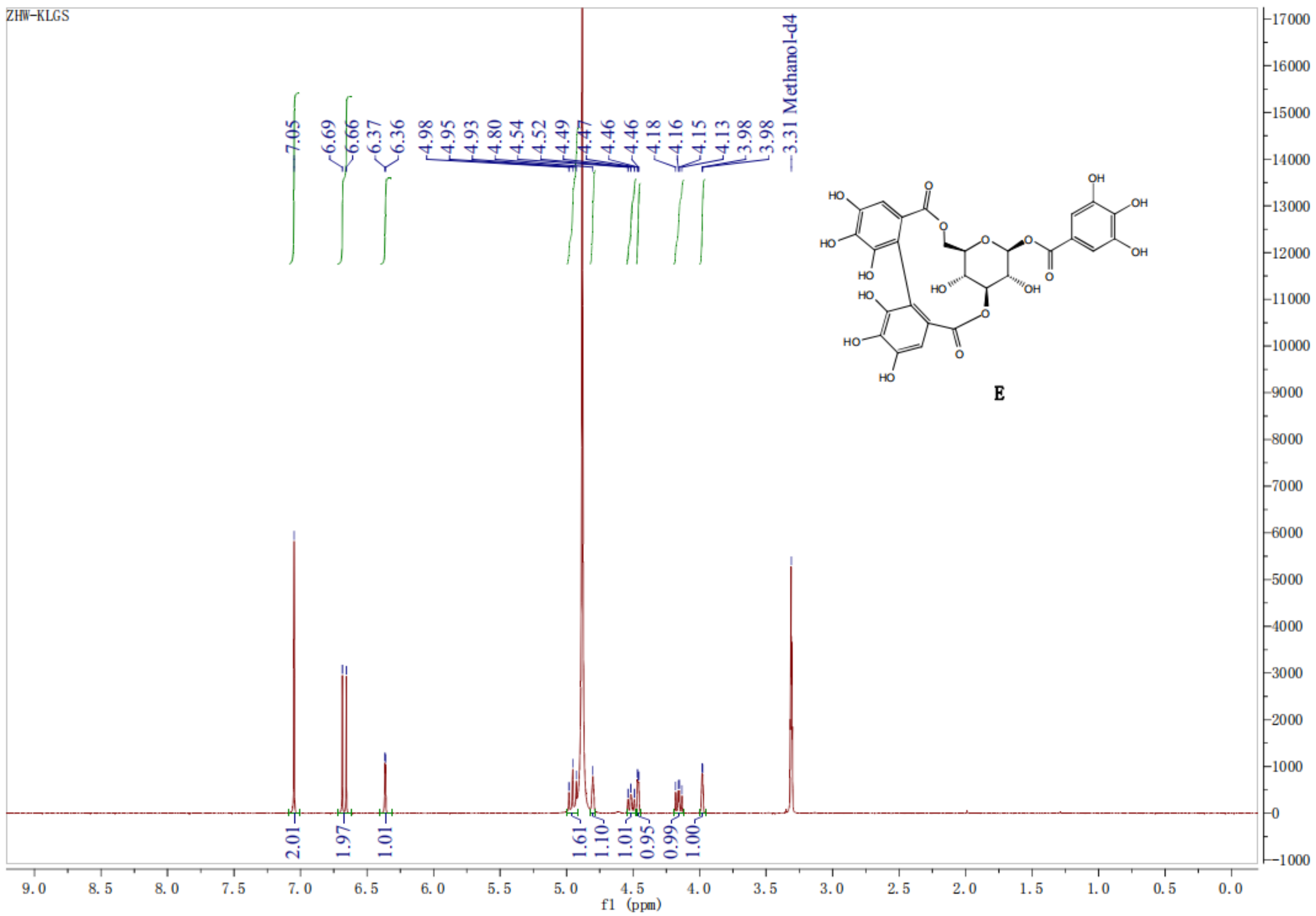

Figure S9 ^{1}H NMR spectrum of E (600 MHz, in MeOH-d_4)

Mass Spectrum SmartFormula Report

Analysis Info

Analysis Name	D:\Data\2018\1220\E.d	Acquisition Date	12/20/2018 10:20:21 AM
Method	tune_pos_low2.m	Operator	BDAL@DE
Sample Name	E	Instrument / Ser#	micrOTOF-Q II 10366
Comment			

Acquisition Parameter

Source Type	ESI	Ion Polarity	Positive	Set Nebulizer	0.3 Bar
Focus	Active	Set Capillary	4800 V	Set Dry Heater	180 °C
Scan Begin	100 m/z	Set End Plate Offset	-500 V	Set Dry Gas	4.0 l/min
Scan End	1000 m/z	Set Collision Cell RF	300.0 Vpp	Set Divert Valve	Waste

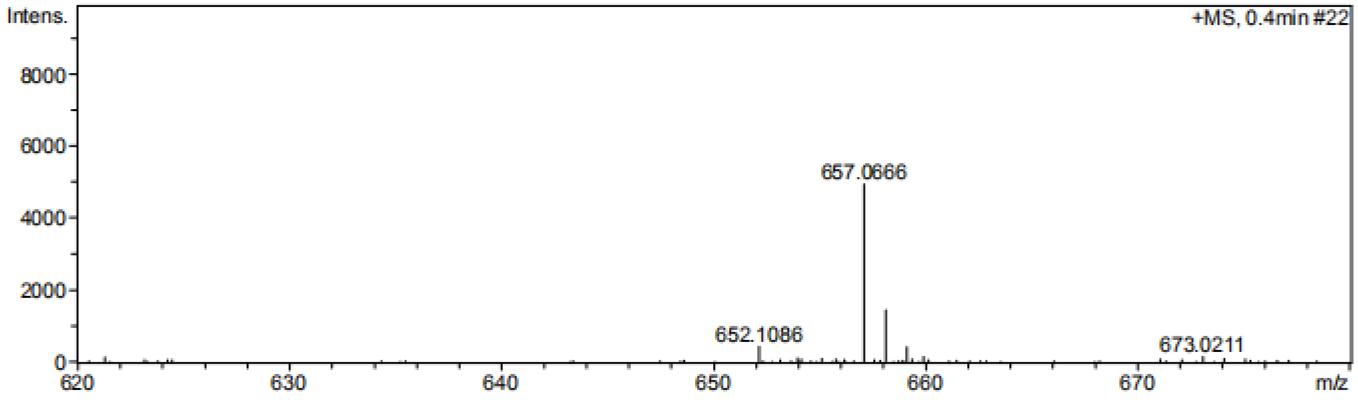

Meas. m/z	Formula	m/z	err [ppm]	mSigma
657.0666	C 27 H 22 Na O 18	657.0698	4.9	4.6

Figure S10 HR-ESI-MS spectrum of E

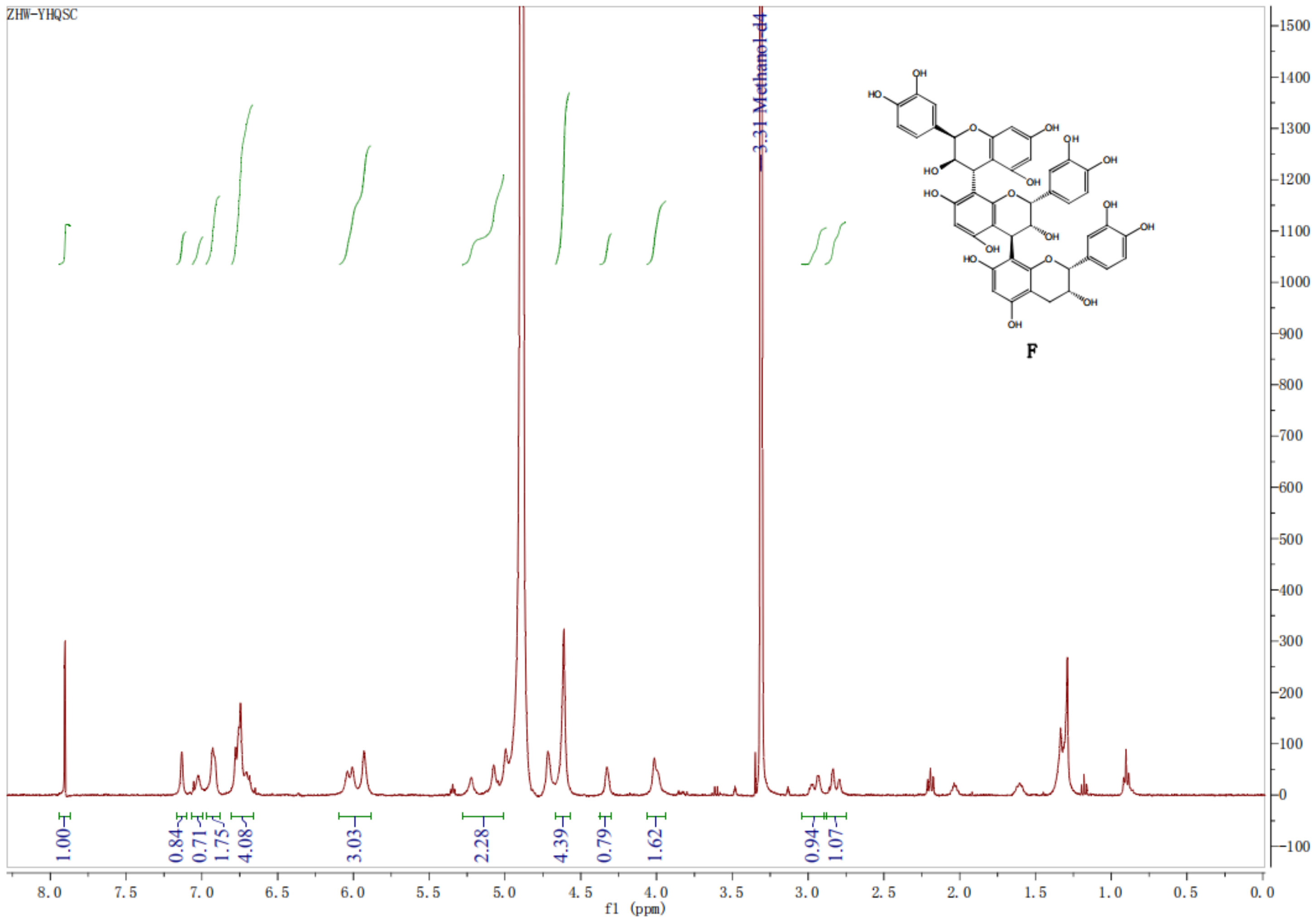

Figure S11 ^{1}H NMR spectrum of F (600 MHz, in MeOH-d_4)

Mass Spectrum SmartFormula Report

Analysis Info

Analysis Name	D:\Data\2018\1220\F..d	Acquisition Date	12/20/2018 12:26:07 PM
Method	tune_pos_wide.m	Operator	BDAL@DE
Sample Name	F	Instrument / Ser#	micrOTOF-Q II 10366
Comment			

Acquisition Parameter

Source Type	ESI	Ion Polarity	Positive	Set Nebulizer	0.5 Bar
Focus	Active	Set Capillary	5500 V	Set Dry Heater	180 °C
Scan Begin	50 m/z	Set End Plate Offset	-500 V	Set Dry Gas	3.0 l/min
Scan End	2000 m/z	Set Collision Cell RF	650.0 Vpp	Set Divert Valve	Waste

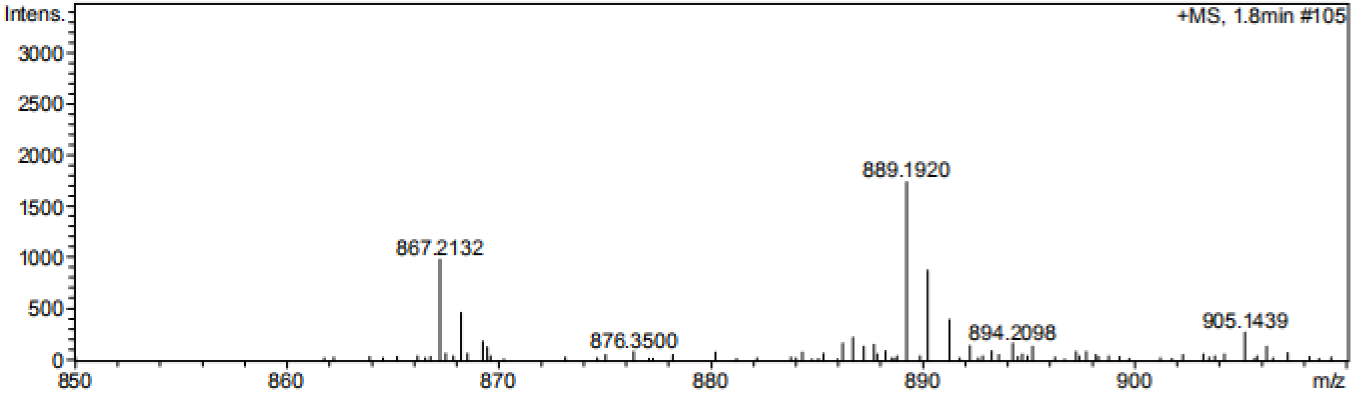

Meas. m/z	Formula	m/z	err [ppm]	mSigma
867.2132	C 45 H 39 O 18	867.2131	-0.1	19.8
889.1920	C 45 H 38 Na O 18	889.1950	3.4	44.1

Figure S12 HR-ESI-MS spectrum of F
